# Hyperphosphataemia, but not hypercalcaemia, predicts cardiovascular risk after kidney transplantation

**DOI:** 10.1093/ckj/sfag024

**Published:** 2026-01-30

**Authors:** Timna Agur, Tali Steinmetz, Maya Haran, Ruth Rahamimov, Shira Goldman, Noam Nagel, Eshcar Meisel, Boris Zingerman, Eviatar Nesher, Benaya Rozen-Zvi

**Affiliations:** Department of Nephrology and Hypertension, Rabin Medical Center, Petah Tikva, Israel; Gray Faculty of Medical and Health Science, Tel Aviv University, Tel Aviv, Israel; Department of Nephrology and Hypertension, Rabin Medical Center, Petah Tikva, Israel; Gray Faculty of Medical and Health Science, Tel Aviv University, Tel Aviv, Israel; Gray Faculty of Medical and Health Science, Tel Aviv University, Tel Aviv, Israel; Department of Nephrology and Hypertension, Rabin Medical Center, Petah Tikva, Israel; Gray Faculty of Medical and Health Science, Tel Aviv University, Tel Aviv, Israel; Department of Transplantation, Rabin Medical Center, Petah Tikva, Israel; Department of Nephrology and Hypertension, Rabin Medical Center, Petah Tikva, Israel; Gray Faculty of Medical and Health Science, Tel Aviv University, Tel Aviv, Israel; Gray Faculty of Medical and Health Science, Tel Aviv University, Tel Aviv, Israel; Department of Nephrology and Hypertension, Rabin Medical Center, Petah Tikva, Israel; Gray Faculty of Medical and Health Science, Tel Aviv University, Tel Aviv, Israel; Department of Nephrology and Hypertension, Rabin Medical Center, Petah Tikva, Israel; Gray Faculty of Medical and Health Science, Tel Aviv University, Tel Aviv, Israel; Gray Faculty of Medical and Health Science, Tel Aviv University, Tel Aviv, Israel; Department of Transplantation, Rabin Medical Center, Petah Tikva, Israel; Department of Nephrology and Hypertension, Rabin Medical Center, Petah Tikva, Israel; Gray Faculty of Medical and Health Science, Tel Aviv University, Tel Aviv, Israel

**Keywords:** cardiovascular disease, hypercalcemia, hyperphosphatemia, kidney transplant recipients, mineral and bone disorder

## Abstract

**Background:**

Kidney transplant recipients (KTRs) face a heightened risk of cardiovascular disease but the impact of post-transplant mineral and bone disorders on this risk remains unclear. This study investigated the association between serum calcium, phosphate and calcium–phosphate (Ca × P) product levels and cardiovascular outcomes.

**Methods:**

In this retrospective cohort study, adult KTRs transplanted between 2005 and 2014 at a large centre were analysed. The primary outcome was major adverse cardiovascular events (MACE). Secondary outcomes included all-cause mortality and a composite of MACE and mortality. Cumulative exposure to abnormal mineral levels was assessed using time-weighted average calculations. Cox proportional hazards models were used to evaluate associations adjusting for confounders, including estimated glomerular filtration rate (eGFR). Mineral abnormalities were analysed both as continuous variables and by quartiles.

**Results:**

The study included 649 KTRs (median follow-up 2943 days), with 109 patients (16.8%) experiencing MACE. Over time, calcium exposure decreased, while phosphate and Ca × P product exposure increased. Hypercalcaemia was not significantly associated with MACE, all-cause mortality or the composite outcome. In contrast, hyperphosphataemia remained significantly associated with an increased risk of MACE {hazard ratio [HR] 1.414 [95% confidence interval (CI) 1.044–1.916]) and the composite outcome [HR 1.353 (95% CI 1.064–.721)] even after adjustment for eGFR. Elevated, Ca × P product levels were similarly associated with increased cardiovascular risk [HR 1.055/mg^2^/dl^2^ (95% CI 1.015–1.096) and HR 1.043/mg^2^/dl^2^ (95% CI 1.013–1.075), respectively].

**Conclusions:**

While hypercalcaemia does not independently predict cardiovascular outcomes post-transplant, sustained hyperphosphataemia and elevated Ca × P product are significant risk factors for adverse cardiovascular events in KTRs.

## Graphical Abstract

**Graphical Abstract:**
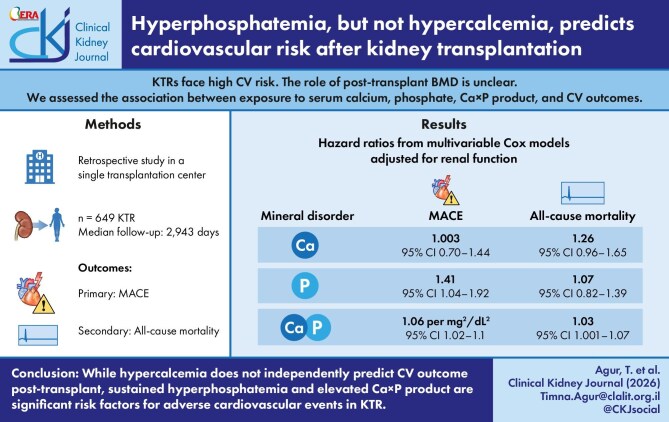

KEY LEARNING POINTS **What was known:**

- Kidney transplant recipients (KTRs) are at an increased risk for cardiovascular disease, yet conventional risk factors do not fully explain this risk.
- Although mineral and bone disorders (MBDs) are common following kidney transplantation, their contribution to cardiovascular risk among KTRs remains uncertain.

**This study adds:**

- This study evaluated the association between serum calcium, phosphate and calcium–phosphate (Ca × P) product levels and cardiovascular outcomes in a cohort of 649 KTRs, assessing cumulative mineral exposure over an 8-year period.
- Elevated calcium levels were not independently associated with increased cardiovascular risk or all-cause mortality.
- Conversely, higher cumulative exposure to phosphate and Ca × P product was independently associated with an increased risk of cardiovascular events, even after adjusting for kidney function, although not with all-cause mortality.

**Potential impact:**

- These findings indicate that post-transplant hypercalcaemia, although prevalent, is not an independent predictor of cardiovascular risk.
- Phosphate-related mineral disturbances may contribute to cardiovascular risk independent of kidney function.
- The results highlight the need for prospective studies to investigate whether targeting hyperphosphataemia with phosphate binders may reduce cardiovascular risk in KTRs.

## INTRODUCTION

Cardiovascular disease (CVD) remains the leading cause of death among kidney transplant recipients (KTRs), with an annual event rate of nearly 5%, significantly higher than that observed in the general population, even after adjusting for conventional cardiovascular risk factors [1]. In both chronic kidney disease (CKD) and the KTR population, traditional risk factors like age, hypertension and diabetes do not fully account for the observed cardiovascular risk, indicating a substantial contribution from non-traditional risk factors [2, 3].

In KTRs, CVD is driven by both atherosclerosis and vascular calcification. The latter is frequently observed in aging, diabetes, dyslipidaemia and CKD. In CKD, vascular calcification affects both the intimal and medial layers of the arterial wall, with medial calcification particularly associated with mineral metabolism disorders [4, 5].

Advanced CKD is marked by secondary hyperparathyroidism, indicated by elevated levels of parathyroid hormone (PTH) and fibroblast growth factor 23 (FGF23), reduced calcitriol, hypocalcaemia and hyperphosphataemia—factors that promote vascular calcification, increased cardiovascular morbidity and further CKD progression [6–10].

While kidney transplantation may partially correct these abnormalities, normalization is often delayed. Persistent hyperparathyroidism affects ≈20% of recipients, even years after transplantation, and is frequently accompanied by ongoing hypercalcaemia and hypophosphataemia and potentially increased cardiovascular risk [11–13]. However, the clinical impact of post-transplant hyperparathyroidism on cardiovascular risk remains controversial [14, 15] and may be affected by factors such as dialysis duration and the severity of mineral imbalances [5, 12, 16–20].

Although kidney transplantation slows the progression of coronary artery calcifications compared with dialysis, it does not fully prevent it; coronary artery calcifications continue to be a strong predictor of cardiovascular events and mortality in KTRs [4, 21–25].

Several studies have examined the association between post-transplant calcium and phosphate levels and cardiovascular outcomes in KTRs, but findings have been inconsistent, ranging from increased mortality risk to no association, or even a U-shaped relationship [5, 12, 16, 18–20, 26–29]. Consequently, the clinical implications of post-transplant calcium and phosphate abnormalities remain uncertain, with limited evidence to guide optimal evaluation and management.

This study aims to assess the association between post-transplant bone mineral disturbances and cardiovascular risk in KTRs.

## MATERIALS AND METHODS

### Patient population and study design

This retrospective cohort study was conducted at the Rabin Medical Center (RMC) and approved by the RMC Institutional Review Board (1062-20-RMC), in accordance with the Declarations of Helsinki and Istanbul. The cohort included all patients ≥18 years of age who underwent kidney transplantation at the RMC between 1 January 2005 and 28 April 2014. Inclusion criteria included a functioning graft at 1-year post-transplantation, at least 2 years of follow-up and six or more serum calcium and phosphate measurements. Patients with multi-organ transplants or cardiovascular events within the first-year post-transplant were excluded. Demographic, clinical and laboratory data were retrieved from electronic medical records. All-cause mortality and cause of death classifications were obtained from the Israel national death registry. Serum calcium and phosphate monitoring was initiated 6 months post-transplant. Only serum calcium and phosphate measurements obtained on the same day were included in the analyses.

The primary outcome was major adverse cardiovascular events (MACE), including non-fatal myocardial infarction (MI), non-fatal cerebrovascular accident (CVA), coronary revascularization, acute coronary syndrome (ACS) hospitalization or cardiovascular death. Secondary outcomes included all-cause mortality and a composite of MACE and all-cause mortality. Events were tracked from 1-year post-transplant until the end of follow-up.

### Immunosuppression protocol and routine monitoring

Standard immunosuppression therapy included tacrolimus, mycophenolate mofetil and prednisolone. Tacrolimus target trough levels were 9–13 ng/ml for the first 3 months and 6–8 ng/ml thereafter, mycophenolate mofetil levels were 1440 mg in the first 2 weeks and 1080 mg thereafter and prednisone was 5 mg since 3 months post-transplantation. Serum calcium and phosphate were routinely measured pre- and post-transplant. Management was at the discretion of the treating nephrologist and was not subject to a formal treatment protocol. Phosphate was supplemented with potassium or sodium phosphate, typically when symptomatic or if serum phosphate dropped below 1.8 mg/dl.

### Statistical analysis

Cumulative exposures to calcium and phosphate were assessed using time-weighted average (TWA), starting 6 months post-transplant. Each value was weighted by the time since the previous measurement and divided by the total follow-up time. The TWA was associated with outcomes based on the year prior to each event. To identify potential hazardous threshold values for mineral abnormalities, analyses were also conducted based on quartiles. Quartiles were derived from the TWA of serum calcium and phosphate, calculated since 6 months after transplantation and updated throughout follow-up. Notably, quartile assignment was time varying, meaning it could change over the follow-up period rather than remaining fixed. Additionally, to account for kidney function, analyses were stratified by estimated glomerular filtration rate (eGFR) quantiles.

Changes over time were analysed using repeated measures analysis of variance. Time-to-event analyses for the occurrence of the first MACE and mortality were performed using a time-varying Cox proportional hazard model with cumulative exposure to calcium, phosphate or Ca × P product as predictors.

Multivariate models were constructed using forward stepwise selection from candidate confounders, with an entry criterion of *P* < .05. In addition to variables demonstrating univariable associations, prespecified covariates based on clinical relevance and prior literature were included in the multivariable models regardless of their univariable *P*-values.

Because of preliminary results, the model was stratified according to sex. The final model encompassed variables such as age, sex, dialysis vintage, donor type (living or deceased), presence of diabetes within the first 6 months post-transplantation, history of ischaemic heart disease prior to transplantation, smoking status, body mass index (BMI), serum albumin and serum PTH level, vitamin D supplement use and eGFR calculated using the Chronic Kidney Disease Epidemiology Collaboration (CKD-EPI) equation and stratified into quantiles (Supplementary Table S1).

## RESULTS

### Patient enrolment and baseline characteristics

**Figure 1:**
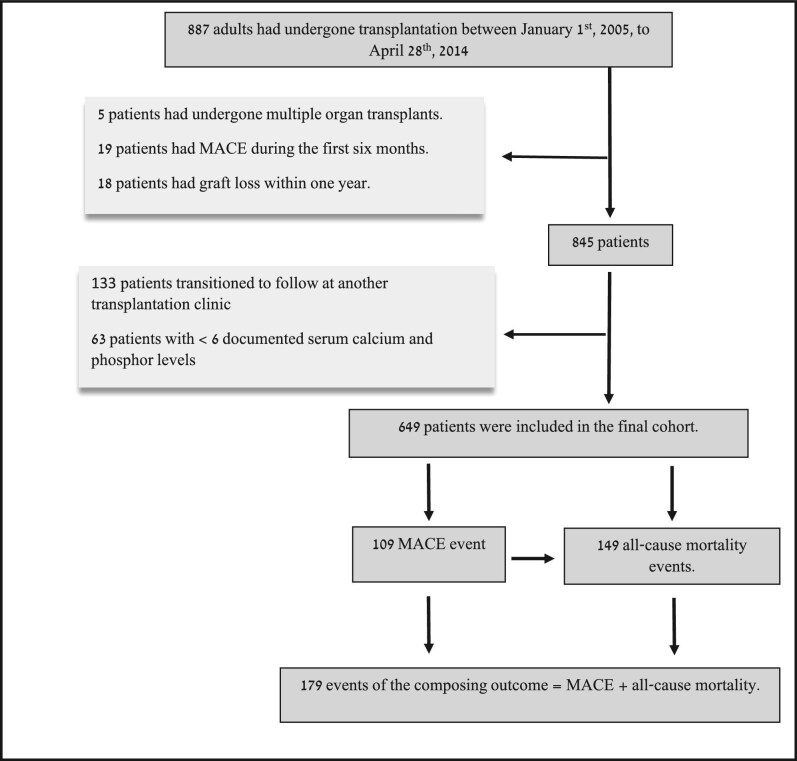
Study flow chart.

Between January 2005 and April 2014, a total of 887 patients underwent kidney transplantation. Among them, 649 recipients met the predefined inclusion and were thus included in this study (Fig. 1). Of these, 32% were female with a mean age of 49.65 ± 14.49 years. The median dialysis vintage was 22 months [interquartile range (IQR) 3–53]. Approximately 59% of the transplantations were from living donors. Pre-transplant, 39% had diabetes and 23% had ischaemic heart disease. The mean eGFR at 3 months post-transplantation was 59.81 ± 20.28 ml/min/1.73 m^2^ (Table 1). The median follow-up duration was ≈8 years (2943 days; IQR 2450–3669). Overall, 59 281 plasma calcium and phosphate measurements were available from 649 patients, with a median of 58 measurements per patient (IQR 38–84).

**Table 1:** Cohort (N=649 patients) characteristics during the first year post-transplant.

Variables	Values
Age (years), mean ± SD	49.65 ± 14.49
Female, *n* (%)	208 (32)
Dialysis vintage (months), median (IQR)	22 (3–53)
Living donor, *n* (%)	381 (58.7)
Recurrent transplantation, *n* (%)	63 (9.7)
eGFR 3 months post-transplant (ml/min/1.73 m^2^), mean ± SD	59.81 ± 20.28
PTH 1 year post-transplant (pg/ml), median (IQR)	116.8 (71.4–201.9)
Serum albumin (g/dl), mean ± SD	4.29 ± 0.35
BMI (kg/m^2^), mean ± SD	26.39 ± 4.89
DM 6 months post-transplant, *n* (%)	251 (38.7)
Hypertension, *n* (%)	430 (66.3)
IHD history, *n* (%)	150 (23.1)
Current smoking, *n* (%)	104 (16)
Statins treatment, *n* (%)	313 (48.2)
RASI, *n* (%)	125 (19.3)
Beta blocker, *n* (%)	257 (44.2)
Calcium supplement, *n* (%)	34 (5.2)
Vitamin D supplement, *n* (%)	68 (10.5)
Bisphosphonate, *n* (%)	8 (1.2)
Calcimimetics, *n* (%)	9 (1.4)

SD: standard deviation; eGFR: estimated by the CKD-EPI equation; IHD: ischaemic heart disease; DM: diabetes mellitus; RAS: renin–angiotensin system.

Patients in the highest calcium quartile (Q4), representing the highest exposure to hypercalcaemia, were older (*P* = .042) and had a greater frequency of deceased-donor transplants (*P* < .001). They showed a non-significant tendency to longer dialysis vintage before transplantation (*P* = .106) and the serum PTH level was higher in this group (<0.001). Use of calcimimetics was uncommon overall (4.3%) but was significantly more frequent in Q4 (*P* = .02) Conversely, patients in the highest serum phosphate quartile (Q4) were younger (*P* = .035), included a higher proportion of women (*P* < .001), had a lower eGFR level (*P* = .019) and had a shorter dialysis vintage prior to transplantation (*P* = .007). Their PTH level was lower compared with patients in other quartiles and the use of calcium supplements was more common (10%; *P* = .009) (Supplementary Table S2).

During follow-up, 109 (17%) patients experienced MACE, 149 (23%) died (all-cause mortality) and 179 (28%) met the composite outcome (Fig. 1). The 109 MACE events were distributed as follows: acute coronary syndrome (30%), percutaneous transluminal coronary angioplasty (24%), MI (17%), cerebrovascular accident (22%) and cardiovascular death (7%) (Supplementary Fig. S1).

### Correlation between cardiovascular risk and calcium exposure

During the follow-up period, a gradual decline in cumulative mean calcium level was observed, decreasing from 9.6 ± 0.49 mg/dl in the first-year post-transplantation to 9.35 ± 0.58 mg/dl by the eighth year. This downward trend in cumulative calcium exposure was consistently observed across all four quartiles throughout the study duration (Supplementary Fig. S2).

No significant association was identified between higher exposure to hypercalcaemia and the risk of MACE whether calcium level was analysed as a continuous variable in univariate or multivariate analysis, [multivariate analysis: HR 1.003 (95% CI 0.701–1.437), *P* = 0.985] or when comparing patients in the highest calcium quartile (Q4) to those in any individual lower quartile or to the combined lower three quartiles [HR 1.064 (95% CI 0.68–1.664), *P* = .787].

**Figure 2:**
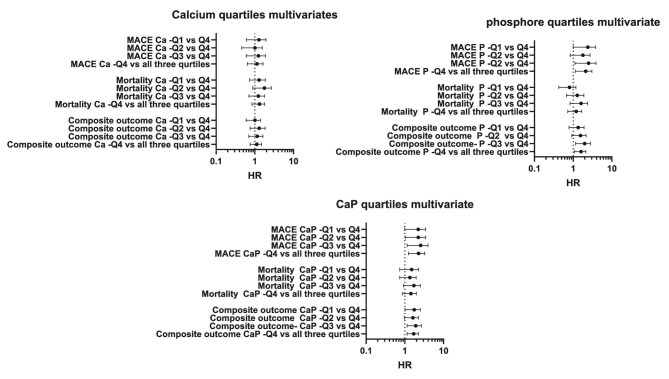
Multivariable analysis of cardiovascular risk by quartiles of cumulative calcium, phosphate and Ca × P product exposure. Calcium exposure was analysed by stratification into quartiles, with Q4 representing the highest level of calcium exposure. The multivariable analysis included adjustment for potential confounders, including age, sex, dialysis vintage, donor type, diabetes, ischaemic heart disease, smoking status, BMI and eGFR (calculated using the CKD-EPI equation and stratified into quintiles). The *x*-axis is presented on a logarithmic scale.

Exposure to elevated calcium levels was also not significantly associated with secondary outcomes, including all-cause mortality and the composite outcome. This was consistent whether calcium was evaluated as a continuous variable [HR for mortality 1.261 (95% CI 0.964–1.65), *P* = 0.09 and HR for composite outcome 1.087 (95% CI 0.812–1.456), *P* = .09] or categorized by quartiles (Q4 versus the combined lower quartiles) [HR for mortality 1.263 (95% CI 0.878–1.815), *P* = .208 and HR for composite outcome 1.086 (95% CI 0.779–1.513), *P* = .626, respectively] (Table 2, Fig. 2, Supplementary Fig. S3).

**Table 2:** Univariate and multivariate analysis of the correlation between cardiovascular risk and calcium, phosphate and Ca × P product exposure.

	Calcium						Phosphate						Ca × P product					
Characteristics	Univariate			Multivariate			Univariate			Multivariate			Univariate			Multivariate		
	HR	95% CI	*P*-value	HR	95% CI	*P*-value	HR	95% CI	*P*-value	HR	95% CI	*P*-value	HR	95% CI	*P*-value	HR	95% CI	*P*-value
MACE																		
Continuous value	1.085	0.758–1.553	0.655	1.003	0.701–1.437	0.985	1.226	0.956–1.572	0.108	1.414	1.044–1.916	0.025	1.037	1.005–1.069	0.022	1.055	1.016–1.096	0.006
1st quartile vs 4th quartile	1.053	0.631–1.754	0.844	1.161	0.674–1.998	0.591	1.709	0.998–2.933	0.051	2.119	1.155–3.891	0.015	1.821	1.085–3.058	0.023	1.988	1.119–3.534	0.019
2nd quartile vs 4th quartile	1.340	0.779–2.304	0.29	0.908	0.517–1.594	0.737	1.337	0.808–2.208	0.258	1.621	0.935–2.809	0.085	1.754	1.065–2.899	0.027	2.020	1.157–3.534	0.013
3rd quartile vs 4th quartile	1.075	0.64–1.808	0.783	1.130	0.662–1.93	0.654	1.678	0.97–2.907	0.064	2.237	1.259–3.984	0.006	1.988	1.155–3.413	0.013	2.299	1.295–4.082	0.004
4th quartile vs all other quartiles	1.144	0.749–1.749	0.533	1.064	0.68–1.664	0.787	1.552	1.025–2.35	0.038	1.961	1.231–3.125	0.005	1.844	1.23–2.765	0.003	2.107	1.328–3.345	0.002
All-cause mortality																		
Continuous value	1.111	0.821–1.503	0.497	1.261	0.964–1.65	0.09	1.667	1.105–1.357	0.004	1.07	0.822–1.394	0.614	1.066	1.036–1.097	0.001	1.033	1.001–1.067	0.045
1st quartile vs 4th quartile	1.070	0.712–1.605	0.747	1.212	0.778–1.89	0.395	1.159	0.754–1.783	0.501	0.742	0.458–1.202	0.226	2.967	1.832–4.808	0.001	1.370	0.812–2.309	0.238
2nd quartile vs 4th quartile	2.188	1.325–3.61	0.002	1.626	0.968–2.732	0.066	1.667	1.045–2.653	0.032	1.179	0.72–1.931	0.514	2.151	1.404–3.289	0.001	1.259	0.792–2	0.331
3rd quartile vs 4th quartile	1.344	0.873–2.07	0.18	1.161	0.748–1.802	0.506	1.751	1.089–2.817	0.021	1.475	0.898–2.421	0.125	2.188	1.404–3.401	0.001	1.597	0.996–2.558	0.052
4th quartile vs all other quartiles	1.111	0.821–1.503	0.497	1.263	0.878–1.815	0.208	1.637	1.139–2.354	0.008	1.136	0.76–1.698	0.534	2.373	1.677–3.358	0.001	1.359	0.915–2.019	0.129
Composite cardiovascular outcome																		
Continuous value	1.247	0.944–1.647	0.12	1.087	0.812–1.456	0.575	1.321	1.089–1.603	0.005	1.353	1.064–1.721	0.014	1.052	1.026–1.08	0.001	1.043	1.013–1.075	0.005
1st quartile vs 4th quartile	1.192	0.802–1.773	0.384	0.951	0.627–1.445	0.815	1.381	0.948–2.012	0.093	1.267	0.826–1.942	0.277	2.110	1.435–3.106	0.001	1.653	1.074–2.545	0.022
2nd quartile vs 4th quartile	1.575	1.041–2.387	0.032	1.224	0.794–1.883	0.36	1.541	1.052–2.257	0.027	1.458	0.966–2.198	0.073	1.916	1.33–2.762	0.001	1.534	1.024–2.294	0.038
3rd quartile vs 4th quartile	1.287	0.868–1.908	0.209	1.100	0.733–1.65	0.646	1.664	1.12–2.469	0.012	1.859	1.218–2.841	0.004	1.965	1.348–2.865	0.001	1.805	1.2–2.717	0.005
4th quartile vs all other quartiles	1.340	0.979–1.836	0.068	1.086	0.779–1.513	0.626	1.521	1.121–2.062	0.007	1.538	1.099–2.154	0.012	1.990	1.48–2.676	<0.001	1.636	1.177–2.273	0.003

IHD: ischaemic heart disease.

Adjustment for time-varying phosphate levels did not reveal any association between calcium exposure and MACE or all-cause mortality (Supplementary Table S3).

### Correlation between cardiovascular risk and phosphate exposure

Throughout the follow-up period, mean phosphate exposure demonstrated an increasing trend, rising from 3.26 ± 0.59 mg/dl in the first-year post-transplantation to 3.51 ± 0.84 mg/dl by the eighth year. This upward trend in cumulative phosphate exposure was consistent across all four quartiles during the study period (Supplementary Fig. S2).

When phosphate levels were analysed as a continuous variable, no significant association with an increased risk of MACE was observed in a univariate analysis [HR 1.226 (95% CI 0.956–1.572), *P* = .108]. However, a statistically significant association emerged in the multivariate analysis [HR 1.414 (95% CI 1.044–1.916), *P* = .025]. Similarly, quartile-based analysis showed that patients in the highest phosphate quartile (Q4) had a significantly increased risk of MACE compared with those in the combined lower three quartiles, in both univariate and multivariate analysis [HR 1.552 (95% CI 1.025–2.35), *P* = .038 and HR 1.961 95% CI 1.231–3.125), *P* = .005, respectively].

When assessing phosphate levels and all-cause mortality, significant associations were found in univariate analysis, whether phosphate was analysed as a continuous variable [HR 1.667 (95% CI 1.105–1.357), *P* = .004) or by comparing Q4 to all lower quartiles combined [HR 1.637 (95% CI 1.139–2.354), *P* = .008]. However, those associations were no longer significant after adjustment in multivariate models [HR 1.07 (95% CI 0.822–1.394), *P* = .614 and HR 1.136 (95% CI 0.76–1.698), *P* = .534, respectively].

Elevated phosphate levels were significantly associated with an increased risk of the composite outcome in univariate analysis, both when assessed as a continuous variable [HR 1.321 (95% CI 1.089–1.603), *P* = .005] and when comparing Q4 to all lower quartiles combined [HR 1.521 (95% CI 1.121–2.062), *P* = .007]. These associations remained significant in a multivariate analysis [HR 1.353 (95% CI 1.064–1.721), *P* = .014 and HR 1.538 (95% CI 1.099–2.154), *P* = .012, respectively] (Table 2, Fig. 2, Supplementary Fig. S3).

After adjustment for time-varying serum calcium, hyperphosphataemia remained significantly associated with MACE but not with all-cause mortality (Supplementary Table S4).

There was no significant interaction between exposure to hyperphosphataemia and hypercalcaemia with respect to cardiovascular risk (MACE) or all-cause mortality (Supplementary Table S4).

### Correlation between cardiovascular risk and Ca × P product exposure

During follow-up period, cumulative mean exposure to Ca × P product demonstrated an increasing trend from 31.21 ± 5.09 mg^2^/dl^2^ in the first-year post-transplantation to 32.9 ± 6.96 mg^2^/dl^2^ by the eighth year. This upward trend in Ca × P product exposure was consistently observed across all four quartiles throughout the follow-up duration (Supplementary Fig. S2).

A significant association was observed between elevated Ca × P product levels and an increased risk of MACE, regardless of the method of analysis. When assessed as a continuous variable, a higher Ca × P product was associated with an increased MACE risk both in univariate [HR 1.037 (95% CI 1.005–1.069), *P* = .022] and multivariate analyses [HR 1.055 (95% CI 1.016–1.096), *P* = .006]. Similarly, when comparing patients in Q4 with those in the combined lower three quartiles, the association remained significant in both the univariate [HR 1.844 (95% CI 1.23–2.765), *P* = .003] and multivariate models [HR 2.107 (95% CI 1.328–3.345), *P* = .002].

In the analysis of the association between elevated Ca × P product levels and all-cause mortality, significant correlations were observed in the univariate analysis, both when exposure to Ca × P product was analysed as a continuous variable [HR 1.066 (95% CI 1.036–1.097), *P* = .001] and when comparing Q4 with the combined lower quartiles [HR 2.373 (95% CI 1.677–3.358), *P* = .001]. However, in multivariate models, these associations were attenuated and reached only borderline statistical significance [HR 1.033 (95% CI 1.001–1.067), *P* = .045 for continuous variables and HR 1.359 (95% CI 0.915–2.019), *P* = .129 for Q4 versus lower quartiles].

Regarding the composite outcome, elevated Ca × P product exposure was significantly associated with an increased risk in the univariate analysis, both when analysed as a continuous variable [HR 1.052 (95% CI 1.026–1.08), *P* = .001] and when comparing Q4 with the lower three quartiles [HR 1.99 (95% CI 1.48–2.676), *P* < .001]. These associations remained statistically significant in a multivariate analysis as well [HR 1.043 (95% CI 1.013–1.075), *P* = .005 for continuous exposure and HR 1.636 (95% CI 1.177–2.273), *P* = .003 for Q4 versus lower quartiles] (Table 2, Fig. 2, Supplementary Fig. S3).

## DISCUSSION

This study evaluated the association between long-term calcium and phosphate disturbances following kidney transplantation and cardiovascular outcomes in 649 KTRs over an 8-year period. While persistent hypercalcaemia was common, it showed no association with MACE, all-cause mortality or the composite outcome. In contrast, prolonged exposure to hyperphosphataemia and elevated Ca × P product levels were significantly associated with increased cardiovascular risk and the composite outcome. These associations remained robust in multivariable models adjusted for multiple confounders, including eGFR quantiles.

Although parathyroid gland involution commonly occurs after kidney transplantation, nearly 50% of recipients continue to present persistent hyperparathyroidism and the associated mineral disturbance 1-year after transplantation [5, 12–14].

In the current study, consistent with previous reports, recipients in the highest hypercalcaemia quartile tended to be older and had a longer pre-transplant dialysis vintage. They also had higher PTH levels and were more frequently treated with calcimimetics [16, 17]. While hypercalcaemia in the context of primary hyperparathyroidism and secondary hyperparathyroidism with CKD has been associated with increased mortality and cardiovascular risk [30–33], evidence regarding the association with cardiovascular outcomes in KTRs remains inconsistent. In line with our findings, Dhingra *et al.* [34] reported no association between serum calcium levels and CVD risk in a community-based cohort with normal eGFR. Furthermore, most studies to date have not demonstrated a significant association between hypercalcaemia and all-cause mortality or graft dysfunction in KTRs [5, 12, 16]. To our knowledge, this is the first study to evaluate the impact of hypercalcaemia exposure on cardiovascular outcomes in KTRs using extended multivariable analysis over a prolonged follow-up period and to report no significant association.

Hypophosphataemia, commonly observed early after transplantation, was not associated with adverse cardiovascular outcomes in our cohort. This aligns with previous studies suggesting that early hypophosphataemia may indicate good graft function and potentially favourable cardiovascular outcomes [5, 26, 35]. Notably, unlike earlier studies that focused on the initial 3 months after transplant, our monitoring began at 6 months. Intriguingly, von Londen *et al.* [26] reported higher rates of graft failure and cardiovascular mortality in patients who did not develop early hypophosphataemia, hypothesizing that early phosphate leakage may suppress FGF23 and PTH levels [6, 18, 36]. This hormonal suppression may underlie the observed protective cardiovascular effect. Management practice varies, however; at our centre, phosphate supplementation is initiated when serum phosphate levels are <1.8 mg/dl, to prevent symptoms. Future studies are needed to clarify whether phosphate supplementation influences cardiovascular risk and to reassess its routine use in this patient population.

The association between hyperphosphataemia and cardiovascular risk for patients with CKD is well established and presents biological plausibility. Hyperphosphataemia has been linked to various cardiovascular complications, including atherosclerosis, vascular calcification and left ventricular hypertrophy, affecting both CKD and non-CKD populations [6–10, 27, 34, 37, 38]. Furthermore, recent evidence suggests a graded, independent correlation between elevated phosphate levels and adverse cardiovascular outcomes, even within the normal range [34, 39]. KTRs with a subsequent decrease in GFR to 15–45 ml/min/1.73 m^2^, are susceptible to CKD metabolic disorders, including CKD–mineral and bone disorder, akin to those observed in non-KTRs with native kidneys at CKD stages 3b–5 (with eGFR <45 ml/min/1.73 m^2^). Consistent with previous studies, our analysis revealed that eGFR was significantly lower in the quartile with the highest phosphate exposure compared with other quartiles [27]. Importantly, our study is the first to demonstrate a significant and independent association between elevated phosphate exposure and increased cardiovascular risk, irrespective of kidney function, whether phosphate levels were analysed as a continuous variable or by quartiles. However, in contrast to previous reports, we did not observe an independent association between elevated phosphate exposure and an increased risk of all-cause mortality in our KTR cohort [19, 20, 27, 28]. While univariate analysis suggested a correlation between hyperphosphataemia, whether assessed as a continuous variable or stratified into quartiles, and the incidence of overall mortality, these associations appeared to be confounded by impaired kidney function. This discrepancy from previous studies may reflect methodological differences, as we assessed cumulative, time-updated mineral exposure over the follow-up and categorized exposure into quartiles [27].

Furthermore, while multivariate analysis revealed only a marginal association between elevated Ca × P product exposure and all-cause mortality, we identified a significant association between higher Ca × P product exposure and both cardiovascular risk and the composite outcome. These associations remained robust across both continuous and quantile-based analyses, reinforcing the potential clinical relevance of Ca × P product exposure in this population.

Elevated Ca × P product exposure may arise from either hypercalcaemia or hyperphosphataemia. In our cohort, Ca × P product was driven primarily by phosphate: increasing hyperphosphataemia exposure showed a strong and positive correlation with increasing Ca × P product levels, whereas hypercalcaemia exposure declined over the follow-up. Consistently, Ca × P product correlated strongly with phosphate rather than calcium, and both higher phosphate exposure and higher Ca × P product exposure were comparably and significantly associated with MACE. These findings support the potential clinical relevance of Ca × P product exposure in this population, even when calcium values are relatively stable.

The observation that individuals in the highest quartiles of both serum phosphate and Ca × P product exposure exhibit a significantly increased cardiovascular risk compared with those in the lower quartiles suggests the existence of a phosphate threshold above which cardiovascular risk is elevated. These findings underscore the need for future studies to investigate the potential benefits of phosphate binders in KTRs with impaired kidney function and hyperphosphataemia as a strategy to reduce cardiovascular risk.

This study presents several limitations. First, it was conducted at a single centre with a predominantly Caucasian patient population, potentially constraining the generalizability of our findings. Second, due to the retrospective design of our study, data on FGF23 were not available, introducing the possibility of residual confounding. Third, mortality ascertainment was based on the Israeli national death registry, which provides nationwide coverage. However, cause-of-death classification may be subject to misclassification, particularly when cardiovascular disease contributes to death but the terminal event is coded as another cause. Consequently, cardiovascular mortality may be underestimated.

Nevertheless, our study has several strengths, including a large cohort size, a time-dependent analysis encompassing a substantial number of intra-individual measurements and complete and long-term follow-up. Although the observational nature of the study precludes the establishment of target values, we posit that given the dearth of randomized controlled trials addressing this issue, our results contribute significantly to the comprehension of cardiovascular risk associated with post-transplant MBD in KTRs.

In summary, post-transplant hypercalcaemia was not identified as an independent predictor of increased cardiovascular risk among KTRs. In contrast, a robust association was observed between higher cumulative exposure to hyperphosphataemia and higher Ca × P product levels and an increased risk of cardiovascular events among KTRs. These associations remained significant even after adjusting for kidney function, suggesting that phosphate-related mineral disturbances may contribute to cardiovascular risk independent of renal function.

### Statement of ethics

This study protocol was reviewed and approved by the local Ethics Committee of the Rabin Medical Center, Israel, approval number RMC-

### Informed consent

Due to the retrospective nature of the research, patient consent was not required according to the Rabin Medical Center ethics committee.

## Supplementary Material

sfag024_Supplemental_File

## Data Availability

Data from this study will be available upon request to the corresponding author.
