# Supplementary material for: Hyperphosphataemia, but not hypercalcaemia, predicts cardiovascular risk after kidney transplantation
Source: Clin Kidney J. 2026 Jan 30;19(4):sfag024. doi: 10.1093/ckj/sfag024 (PMC13069486; doi:10.1093/ckj/sfag024)

**Supplementary Materials**

1) Supplementary Tables

**1.1) Supplementary Table S1-** Multivariate coefficients

**1.2) Supplementary Table S2 –** Cohort characteristics during the first year post-transplantation, stratified by quartiles of Calcium, Phosphate and Ca⋅P product exposure

**1.3) Supplementary Table S3:** correlation between cumulative calcium exposure by quartiles and cardiovascular outcomes in univariate, multivariate analysis and

multivariate adjusted to time varying phosphate.

**1.4) Supplementary Table S4:** correlation between cumulative phosphor exposure by quartiles and cardiovascular outcomes in univariate, multivariate analysis and

multivariate adjusted to time varying calcium

2) Supplementary Figures

**2.1) Supplementary Figure S1-** Distribution of MACE events during the study follow-up period

**2.2) Supplementary Figure S2 -** Cumulative exposure to Calcium (A) and Phosphate (B) over the study period, stratified by quartiles of exposure. (C) Correlation between elevated Ca⋅P product levels and hyperphosphatemia exposure

**2.3) Supplementary Figure S3 -** Univariate and multivariable analyses of the association between cardiovascular risk and continuous measures of calcium, phosphate, and calcium–phosphate product exposure

Calcium exposure was analyzed both as a continuous variable. The multivariable analysis included adjustment for potential confounders, including age, sex, dialysis vintage, donor type, diabetes, IHD, smoking status, BMI, eGFR which was calculated using the CKD-EPI equation and stratified into quintiles.

HR, hazard ratio; CI, confidence interval; eGFR, estimated glomerular filtration rate; BMI, body mass index; IHD, ischemic heart disease; The X axis is presented on a logarithmic scale

**Supplementary Table S1- Multivariate coefficients**

**
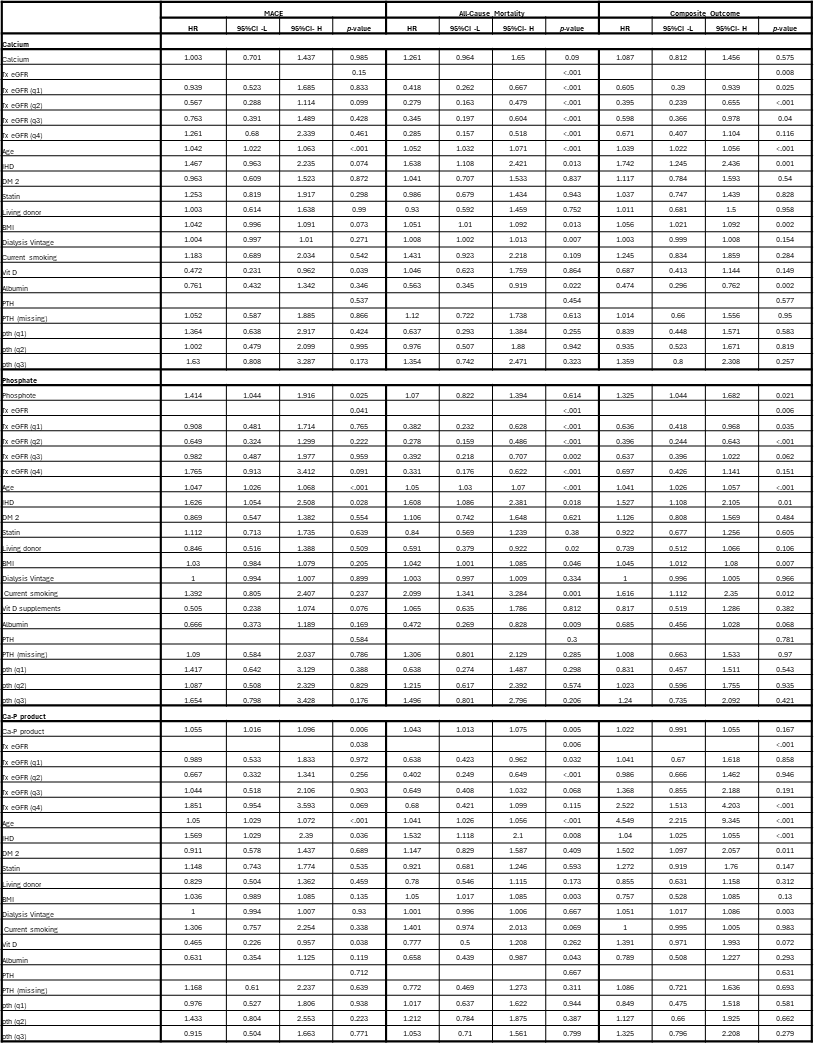
**

Data are mean (SD) or n (%). eGFR, estimated glomerular filtration rate estimated by CKD-EPI equation; BMI, body mass index; IHD, ischemic heart disease; DM, Diabetes Mellitus

**Supplementary Table S2 -** Cohort characteristics during the first year post-transplantation, stratified by quartiles of Calcium, Phosphate and Ca⋅P product exposure


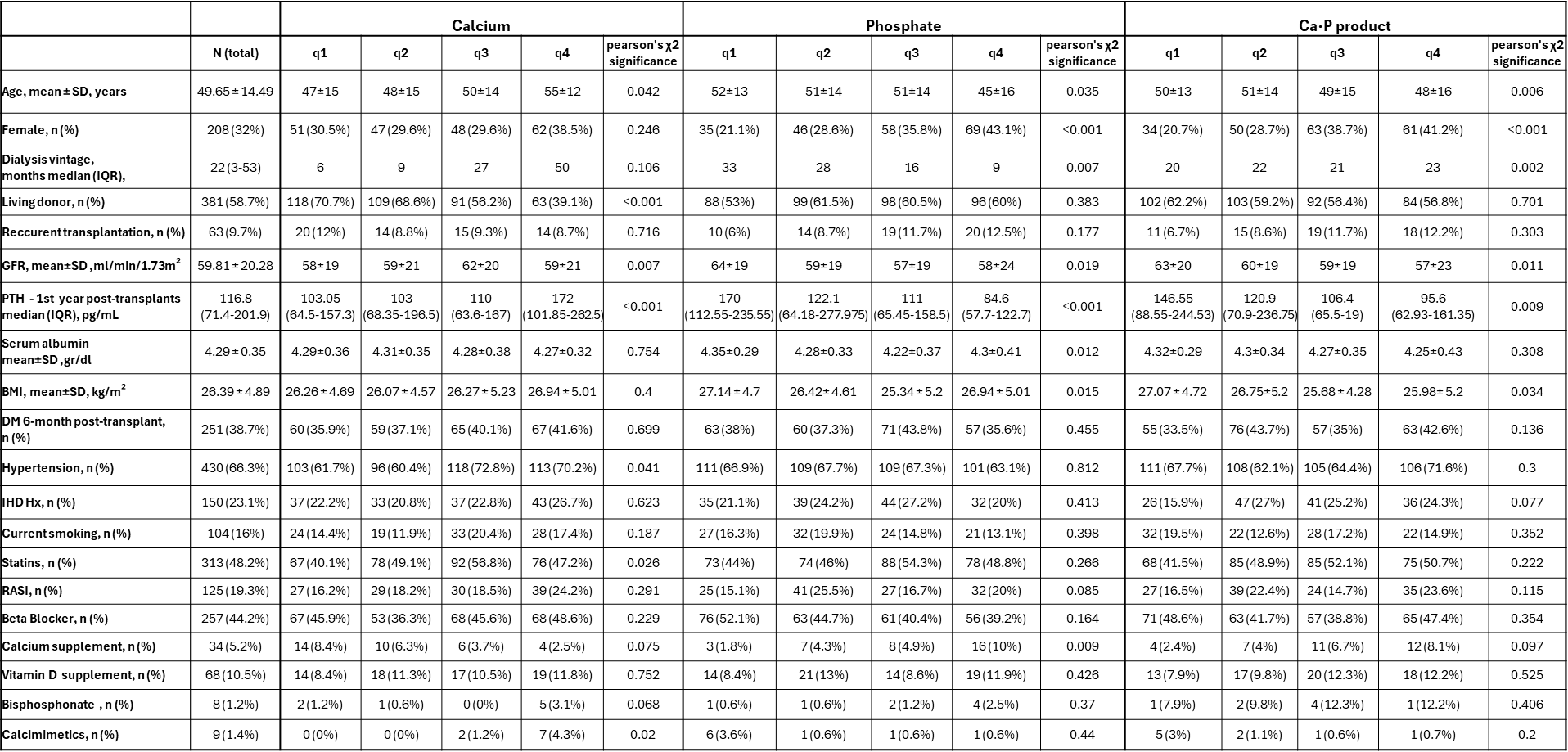


eGFR, estimated glomerular filtration rate estimated by CKD-EPI equation; BMI, body mass index; IHD, ischemic heart disease; DM, Diabetes Mellitus; MACE, Major Adverse Cardiovascular Event

**Supplementary Table S3:** correlation between cumulative calcium exposure by quartiles and cardiovascular outcomes in univariate, multivariate analysis and

multivariate adjusted to time varying phosphate.

^a^

^
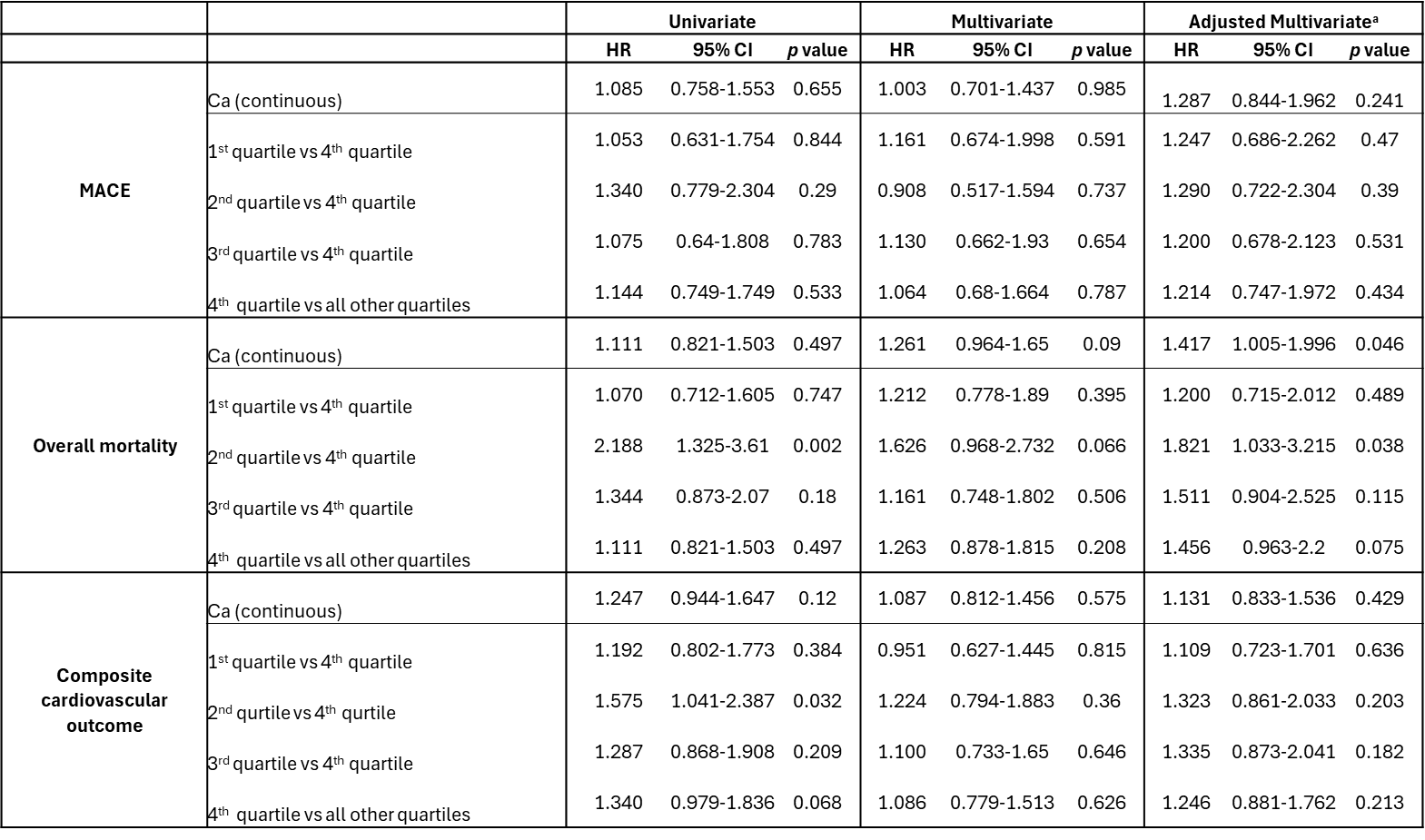
a^ multivariate adjusted to time varying phosphate

**Supplementary Table S4:** Correlation between cumulative phosphor exposure by quartiles and cardiovascular outcomes in univariate, multivariate analysis and multivariate adjusted to time varying calcium


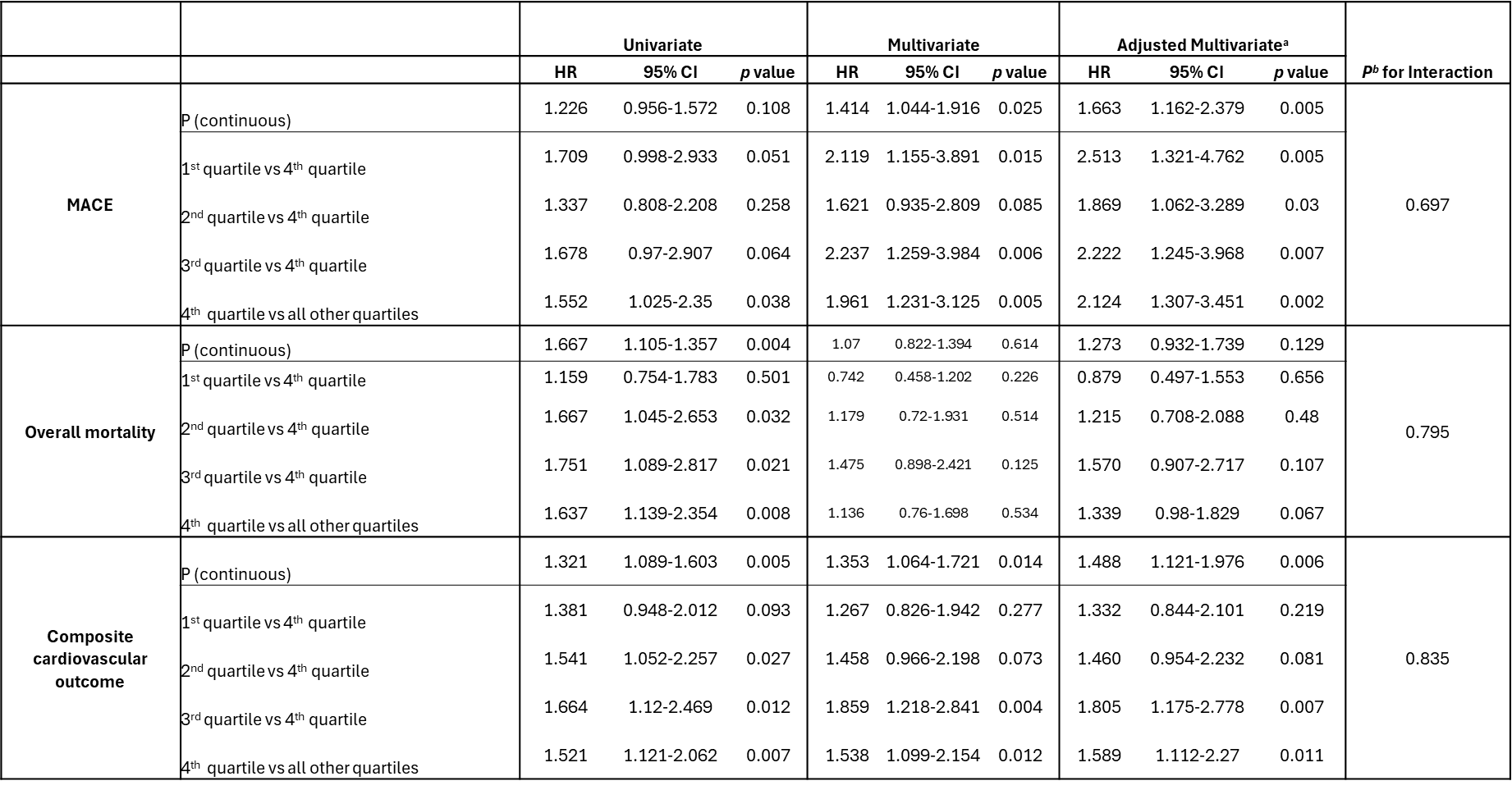


^a^ multivariate adjusted to time varying calcium

^b^ *P* interaction between exposure to hyperphosphatemia and hypercalcemia with respect to cardiovascular risk (MACE) or all-cause mortality

**Supplementary Figure S1-**

**Distribution of MACE events during the study follow-up period**


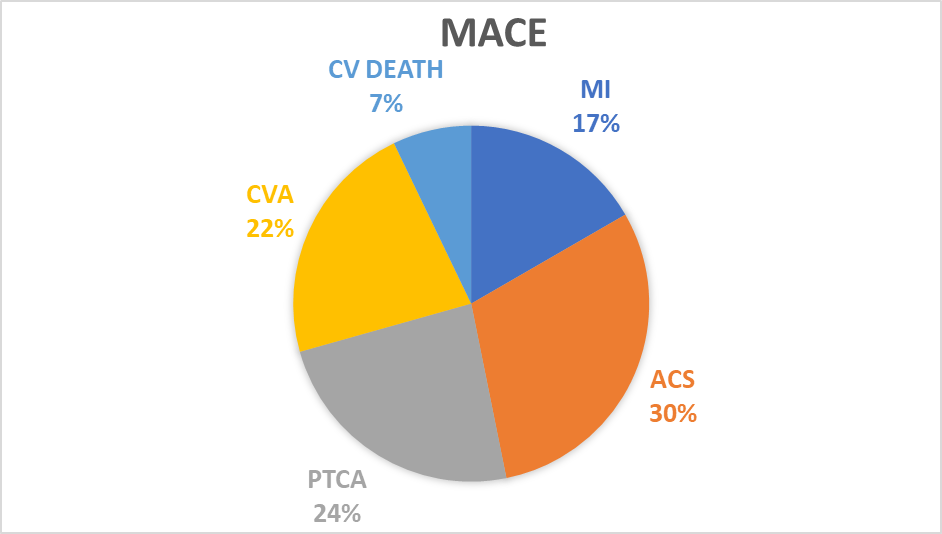


**Supplementary Figure S2-**

Cumulative exposure to Calcium (A) and Phosphate (B) over the study period,

stratified by quartiles of exposure.

C) Correlation between elevated Ca⋅P product levels and hyperphosphatemia

exposure


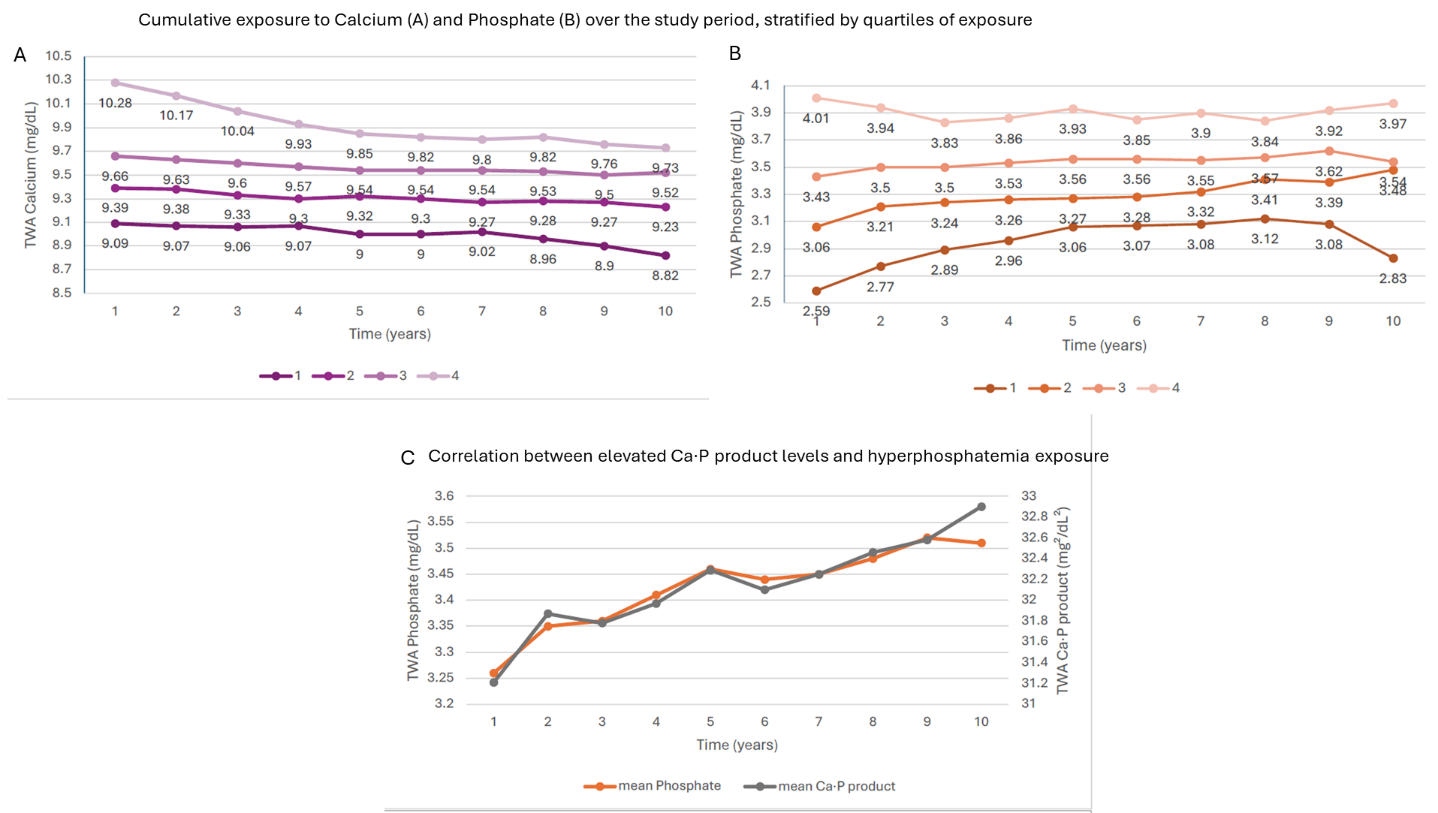


**Supplementary figure S3** – **Univariate and multivariable analyses of the association between cardiovascular risk and continuous measures of calcium, phosphate, and calcium–phosphate product exposure**


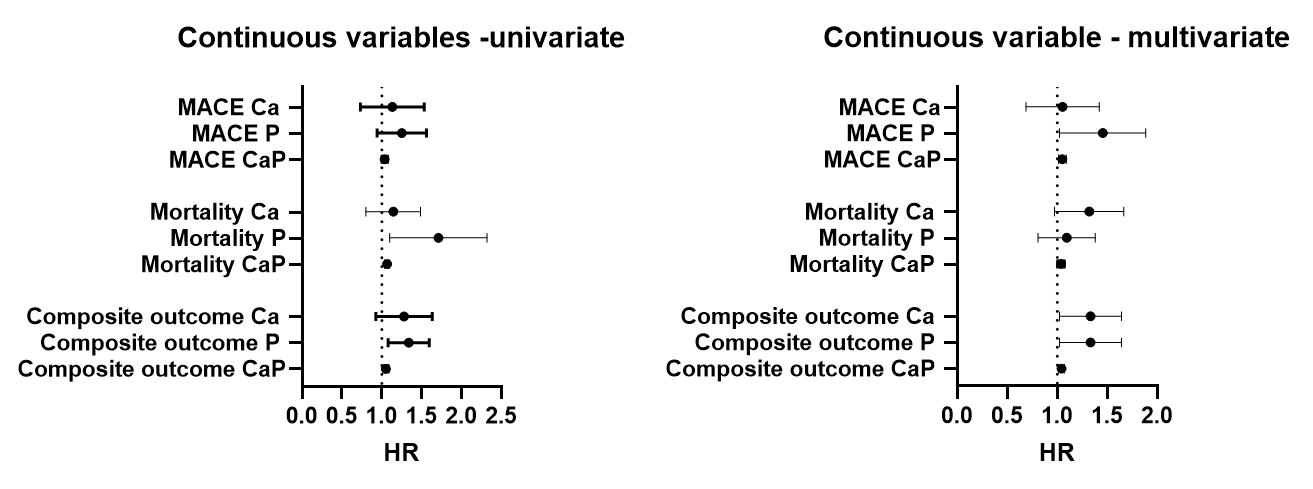

Supplement: sfag024_Supplemental_File [file sfag024_supplemental_file.docx]
